# Maternal HBsAg carriers and pregnancy outcomes: a retrospective cohort analysis of 85,190 pregnancies

**DOI:** 10.1186/s12884-020-03257-4

**Published:** 2020-11-25

**Authors:** Yulong Zhang, Jiacheng Chen, Tingting Liao, Siwen Chen, Jianying Yan, Xiaoqian Lin

**Affiliations:** Department of Obstetrics and Gynecology, Fujian Provincial Maternity and Children’s Hospital, Affiliated Hospital of Fujian Medical University, Fuzhou, 350001 Fujian China

**Keywords:** HBV infection, Adverse pregnancy outcomes

## Abstract

**Background:**

Nowadays, a positive HBV carrier status is common among pregnant women, especially in endemic areas (such as China), little is known about the impact of maternal HBV infection on the risk of adverse pregnancy outcomes. Pregnant women with HBV infection often develop obstetric complications, such as pregnancy-induced hypertension (PIH) syndrome, postpartum hemorrhage, and gestational diabetes mellitus (GDM), and their infants often exhibit neonatal complications.

**Methods:**

This study undertook a retrospective cohort analysis to explore the association of HBV carrier status with adverse pregnancy outcomes. A cohort of 85,190 women including 9699 HBsAg-positive and 73,076 HBsAg-negative pregnancies was retrospectively analyzed.

**Results:**

It’s found that HBsAg-positive pregnancies may result in higher risk of various maternal outcomes such as ICP (OR 3.4,95%CI 2.80 to 4.13), postpartum hemorrhage (OR 1.16,95%CI 1.00 to 1.34). Interestingly, there was a decreased risk of Preeclampsia (OR 0.91,95%CI 0.87 to 0.96), premature rupture of membrane (OR 0.91,95%CI 0.87 to 0.96) and gestational hypertension (OR 0.828,95%CI 0.701 to 0.978). And in vaginal delivery subgroup analysis, It’s found that the HBsAg-positive group had a higher risk of placental abruption (OR, 1.44; 95% CI, 1.16–1.79).

**Conclusions:**

The present results suggest that compared with HBV positive pregnancies were more likely to be ICP and postpartum hemorrhage. HBV-positive pregnant women underwent vaginal delivery were more likely to have placental abruption and premature birth compared with HBV-negative women. Obstetricians should be aware of ICP, postpartum hemorrhage, placental abruption and premature birth in HBV-positive pregnant women.

## Background

Chronic hepatitis B virus (HBV) infection is an important global health problem. Up to 600,000 of the 350–400 million carriers of HBV worldwide die annually of chronic hepatitis B (CHB)-related disease [1]. Women of childbearing age who are infected with HBV tend to be in the immune-tolerant or immune-active phases of chronic HBV infection and have high levels of viremia. The prevalence of HBV infection among these women may be as high as 0.4% in the USA [2] and 2–8% in China [3–5]. Most pregnant women with HBV infection are chronic carriers of the virus, which can be detected by the presence of the hepatitis B surface antigen (HBsAg) in serum.

Although a positive HBV carrier status is common among pregnant women, especially in endemic areas (such as China), little is known about the impact of maternal HBV infection on the risk of adverse pregnancy outcomes. Pregnant women with HBV infection often develop obstetric complications, such as pregnancy-induced hypertension (PIH) syndrome, postpartum hemorrhage, and gestational diabetes mellitus (GDM), and their infants often exhibit neonatal complications. A higher prevalence of preterm delivery in women with chronic HBV infection was reported in several epidemiological studies [6, 7]. intrahepatic cholestasis of pregnancy (ICP) is a pregnancy-specific liver disorder that typically commences in the late-second or third trimester and resolves within 48 h after delivery. Several studies have mentioned an association between ICP and hepatitis C virus (HCV) infection [8]. Locatelli et al. found that women with HCV antibody positivity had a significantly increased incidence of cholestasis compared with those without HCV antibodies (15.9% versus 0.8%, *P* < 0.001) [9]. HBV infection may affect trophoblast cells, which play an important role in placental development [10, 11]. Two recent studies reported an independent association between chronic HBV infection and GDM [12, 13], which is in disagreement with our results. Therefore, additional studies are required to elucidate this issue. Preeclampsia (which is characterized by multilevel maternal endothelial dysfunction) may be triggered by or result from an imbalance between angiogenic, antiangiogenic, and proangiogenic factors, e.g., vascular endothelial growth factor [8, 14]. Ahmed et al. reported that women who were seropositive for HBsAg had a higher risk of preeclampsia compared with women who were seronegative for HBsAg [15]. Conversely, KLB R et al. found no association between maternal HBV infection and preeclampsia [6, 16]. Lao et al. reported that the incidence of gestational diabetes was significantly increased in the HBsAg-positive group.

Moreover, the relationship between HBsAg-positive and adverse pregnancy outcomes remains controversial. Most published studies used data from countries where the prevalence of HBsAg positivity during pregnancy was low. Besides, the outcomes may not be applicable to higher risk countries and regions. Therefore, we performed this retrospective hospital-based cohort study to explore the association between HBV carrier status and adverse pregnancy outcomes.

## Method

### Patients and grouping

**Fig. 1:**
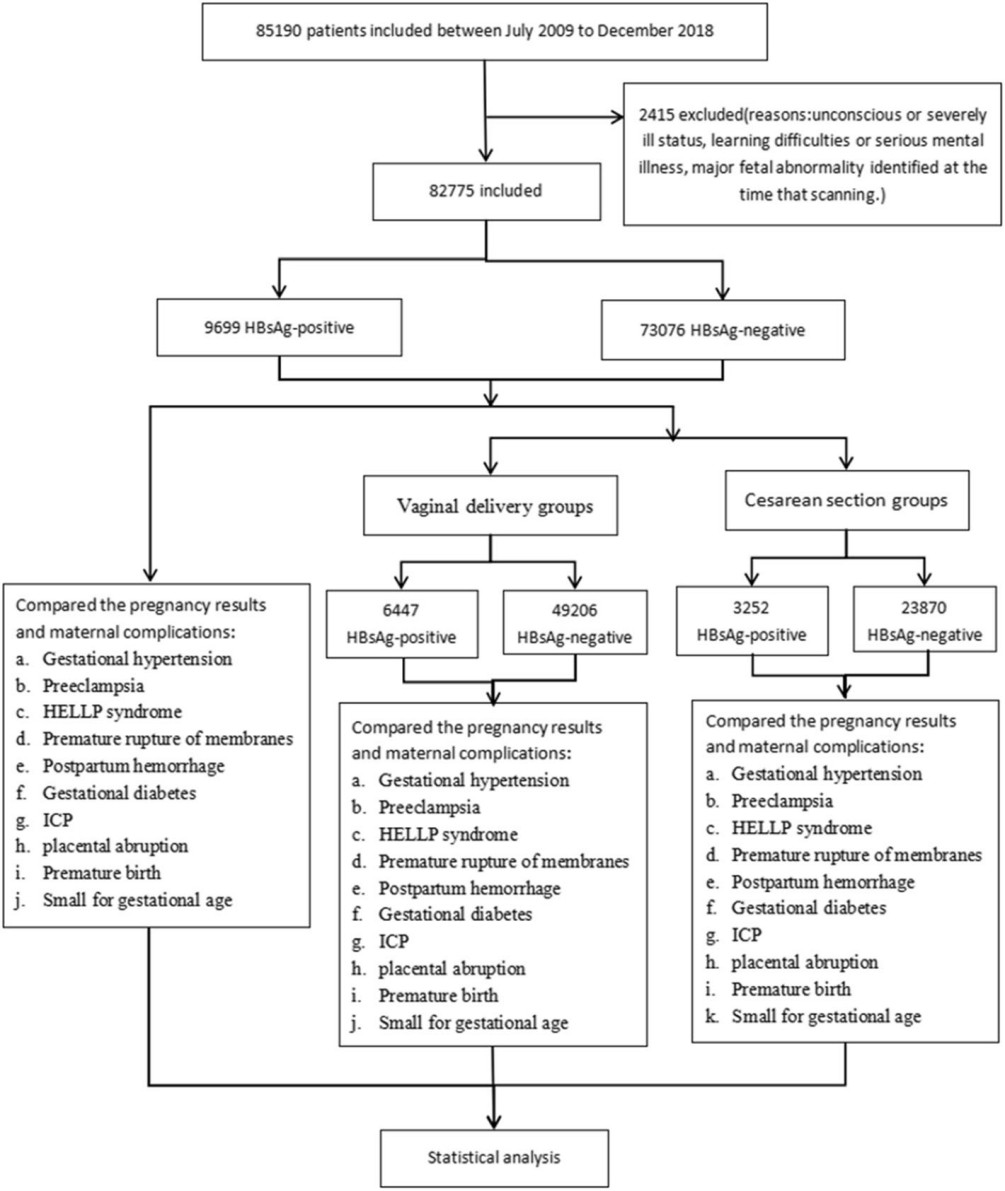
Flow chart of study from total results to the final inclusion or exclusion

All women who were followed at the Fujian Provincial Maternity and Children’s Hospital, China, between July 2009 and December 2018 for singleton pregnancies carried to 24 completed weeks of gestation or beyond were included in this retrospective and consecutive cohort study. A total of 85,190 pregnant women were reviewed and screened, of these, 2415 subjects were excluded: due to unconscious or severely ill status, learning difficulties or serious mental illness, major fetal abnormality identified at the time that scanning. Finally, The cohort included 85,190 women: 9699 HBsAg-positive and 73,076 HBsAg-negative pregnancies. HBV infection was defined as HBsAg seropositivity. The study group consisted of all pregnancies with maternal HBV infection, as demonstrated by a positive HBsAg status on antenatal screening, while the control group consisted of the remainder of the obstetric population. The study was legally approved by the institutional ethics committee of Fujian Provincial Maternity and Children’s Hospital and conducted in accord with the guidelines of the Declaration of Helsinki, and the rights of all participants were protected. Fig. 1 shows a flow chart of study from total results to the final inclusion or exclusion. (Fig. 1).

### Inclusion and exclusion criteria

The inclusion criteria of this trial were as follows: age ≥ 18 years, singleton pregnancy, and maternal HBV infection (as reflected by a positive HBsAg status on antenatal screening). The exclusion criteria were as follows: unconsciousness or a severely ill status, learning difficulties or serious mental illness, and major fetal abnormalities identified at the time of scanning but we were not excluded the subjects who were vaccinated with Hepatitis B vaccine so it can lead to HbsAg positive for approximately 14 days after the vaccine has been given.

### Outcome measurements

We reviewed the records of 82,775 women who had delivered their infants during the study period and recorded the following parameters: pregnancy and delivery characteristics, including gestational age (years; determined from the fetal crown–rump length), gestational age (weeks), gravidity, parity, mode of delivery, neonatal weight, placental weight, and amount of bleeding; and pregnancy outcomes and maternal complications, including gestational hypertension, premature rupture of membranes, postpartum hemorrhage, GDM, intrahepatic cholestasis of pregnancy (ICP), placental abruption, premature birth, HELLP syndrome, abortion, and small-for-gestational-age fetal status.

The diagnostic criteria for GDM were based on the National Health and Family Planning Commission of the People’s Republic of China guidelines. When the 75 g OGTT results met or exceeded the following plasma glucose levels at the noted time-points, the women were diagnosed with GDM: 0 h, 5.1 mmol/L; 1 h, 10.0 mmol/L; and 2 h, 8.5 mmol/L. A 75 g OGTT was performed between the 24th and 28th weeks of gestation for all pregnant women who had not previously been diagnosed with diabetes. Hypertension in pregnancy was defined as a systolic blood pressure of at least 140 mmHg or a diastolic blood pressure of at least 90 mmHg. Using semi-quantitative urine dipsticks, proteinuria of at least 1+ in the presence of hypertension without evidence of urinary tract infection was considered significant. HDPs were classified as: gestational hypertension, chronic hypertension, pre-eclampsia superimposed on chronic hypertension, pre-eclampsia or eclampsia. The diagnosis of PPROM was confirmed by well-established clinical and/or biological diagnostic procedures: the visualization of amniotic fluid passing from the cervical canal and pooling in the vagina, a basic pH test of vaginal fluid, or arborization (ferning) of dried vaginal fluid identified under microscopic evaluation. ICP diagnosis was carried out based on new onset pruritus with a total bile acid level > 10 μmol/l without any additional liver diseases. Small-for-gestational age was defined as neonatal birthweight below the 10th percentile for gestational age. HELLP syndrome was specified by hemolysis (serum LDH > 600 IU/L; bilirubin > 1.2 mg/dL; presence of schistocytes in peripheral blood), elevated liver enzymes (serum ALT and/or AST > 70 IU/L) and thrombocytopenia (platelet count < 100,000/mm3). The diagnosis of placental abruption was based on clinical findings of abdominal pain, vaginal bleeding, uterine contractions, fetal distress and vital sign abnormalities. We excluded women with multiple gestation and gestational age less than 20 weeks.

### Statistical analyses

SPSS 19.0 (SPSS Inc., Chicago, IL) was used to perform the statistical analyses. Continuous data (e.g. age and operating time) are presented as means  ±   standard deviation (SD) and were analyzed using an independent t-test or a nonparametric test (Kruskal–Wallis test). Dichotomous data (e.g. sex and age group) are presented as percentages and were compared between the two groups using the χ^2^ test or a nonparametric test (Fisher’s exact test). Significance was set at P   < 0 .05. In the outcome analyses, relative risks and absolute risk differences with 95% confidence intervals (95% CI) were calculated for dichotomous outcomes using Fisher’s exact test.

## Results

### Baseline characteristics

The patient and pregnancy characteristics are presented in Table 1. Women with singleton pregnancies underwent routine examinations at their hospital. The patient characteristics included: maternal age; cigarette smoking during pregnancy; history of chronic hypertension or diabetes mellitus; family history of PE (Preeclampsia) in the mother of the patient; obstetric history, including parity (parous or nulliparous in the case of presence/absence of previous deliveries at or after 24 weeks of gestation, respectively); previous pregnancy with PE; gestational age at delivery; and the baby’s birthweight. There were no differences in mean maternal age or gestational age between the study group and control group. In contrast, significant differences were found for gravidity (the proportion of nulliparous women was higher in the control group (34.26% versus 38.42%)). However, no significant differences in parity, mode of delivery, and birth weight were found between the two groups. The routes of HBV transmission include sexual contact, parenteral drug use with shared paraphernalia, tattoos, piercings, acupuncture, and needlestick injuries in the health-care setting. In countries where HBV is endemic, most infections are acquired through perinatal exposure.

**Table 1:** Baseline characteristics of the included patients^a^

	HBV-positive (*n* = 3252)	HBV-negative (*n* = 23,870)	*P* value	OR
Maternal age (yrs, mean ± SD)	30.33 ± 4.50	30.28 ± 4.45	*P* = 0.30	
Gestational age (weeks, mean ± SD)	38.18 ± 2.96	38.17 ± 3.51	*P* = 0.76	
Gravidity				
1	3323 (34.26%)	28,076 (38.42%)	*P* < 0.001	0.84 (0.80,0.87)
> 1	6376 (65.74%)	45,000 (61.58%)	*P* < 0.001	1.20 (1.15,1.25)
Parity				
1	82 (0.85%)	760 (1.04%)	*P* = 0.07	0.81 (0.65,1.02)
> 1	9617 (99.15%)	72,316 (98.96%)	*P* = 0.07	1.23 (0.98,1.55)
Mode of delivery				
Instrumental vaginal	1903 (19.62%)	14,009 (19.17%)	*P* = 0.29	1.03 (0.98,1.09)
Cesarean section	3380 (34.85%)	24,729 (33.84%)	*P* = 0.05	1.05 (1.00,1.09)
Vaginal delivery	4373 (45.09%)	33,907 (46.40%)	*P* = 0.01	0.95 (0.91,0.99)
Regional analgesia	43 (0.44%)	431 (0.59%)	*P* = 0.07	0.75 (0.55,1.03)
Birth weight(g)				
< 3000	2855 (29.44%)	20,571 (28.15%)	*P* = 0.008	1.06 (1.02,1.122)
3000–3499	4026 (41.51%)	30,721 (42.04%)	*P* = 0.32	0.98 (0.94,1.02)
3500–3999	2231 (23.00%)	17,085 (23.38%)	*P* = 0.41	0.98 (0.93,1.03)
≥ 4000	388 (6.05%)	2718 (6.43%)	*P* = 0.17	1.08 (0.97,1.20)

### Maternal HBV-positive

Table 2 shows that HBV-positive during pregnancy increased the risk of some maternal outcomes, including ICP (OR, 3.4; 95% CI, 2.80–4.13), and postpartum hemorrhage (OR, 1.16; 95% CI, 1.00–1.34). Interestingly, a decreased risk of preeclampsia (OR, 0.91; 95% CI, 0.87–0.96), premature rupture of membranes (OR, 0.91; 95% CI, 0.87–0.96), and gestational hypertension (OR, 0.828; 95% CI, 0.701–0.978) was observed in this group. There were no significant differences between the two groups regarding placental abruption (OR, 1.17; 95% CI, 0.98–1.4), preterm labor (OR, 1.17; 95% CI, 0.98–1.40), small-for-gestational-age status (OR, 0.93; 95% CI, 0.84–1.04), GDM (OR, 0.8; 95% CI, 0.67–0.96), and HELLP syndrome (OR, 0.78; 95% CI, 0.31–1.97).

**Table 2:** The association between HBV positive pregnancies and outcomes

	HBV-positive (*n* = 3252)	HBV-negative (*n* = 23,870)	*P* value	OR
Gestational hypertension	157 (1.62%)	1421 (1.94%)	*P* = 0.028	0.83 (0.70,0.98)
Preeclampsia	139 (1.43%)	1302 (1.78%)	*P* = 0.014	0.80 (0.67,0.96)
HELLP syndrome	5 (0.05%)	48 (0.07%)	*P* = 0.605	0.78 (0.31,1.97)
P*PROM*	2577 (26.57%)	20,776 (28.43%)	*P* < 0.001	0.91 (0.87,0.96)
Postpartum hemorrhage	218 (2.25%)	1424 (1.95%)	*P* = 0.047	1.16 (1.00,1.34)
GDM	1663 (17.15%)	11,982 (16.40%)	*P* = 0.062	1.06 (1.00,1.12)
ICP	149 (1.54%)	334 (0.46%)	*P* = 0.001	3.40 (2.80,4.13)
placental abruption	140 (1.44%)	905 (1.24%)	*P* = 0.089	1.17 (0.98,1.40)
Premature labor	1158 (11.94%)	8424 (11.53%)	*P* = 0.234	1.04 (0.97,1.11)
Small for gestational age	392 (0.22%)	3165 (0.19%)	*P* = 0.192	0.93 (0.84,1.04)

**Abbreviations:** *GDM* Gestational diabetes, *ICP* Intrahepatic cholestasis of pregnancy, *PPROM* Preterm premature rupture of the membrane

### The association between HBV positive pregnancies and pregnancy outcomes of vaginal delivery

As shown in Table 3, the HBsAg-positive group had a higher risk of postpartum hemorrhage (OR, 1.19; 95% CI, 1.01–1.39), ICP (OR, 3.73; 95% CI, 2.84–4.90), placental abruption (OR, 1.44; 95% CI, 1.16–1.79), and premature birth (OR, 1.44; 95% CI, 1.16–1.79). A significant difference in ICP was also detected between the two groups. The incidence of premature rupture of membranes was significantly lower in HBV carriers (OR, 0.88; 95% CI, 0.83–0.93), suggesting that infection with HBV protects against this outcome. However, small-for-gestational-age status (OR, 1.12; 95% CI, 0.98–1.28), gestational hypertension (OR, 0.83; 95% CI, 0.67–1.04), HELLP syndrome (OR, 1.27; 95% CI, 0.15–10.57), and gestational diabetes (OR, 1.07; 95% CI, 0.99–1.14) were not significantly different between the two groups.

**Table 3:** Gestational complications in Vaginal delivery

	HBV-positive (*n* = 3252)	HBV-negative (*n* = 23,870)	*P*	OR
Gestational hypertension	88 (1.30%)	804 (1.41%)	*P* = 0.106	0.83 (0.67, 1.04)
Preeclampsia	49 (0.76%)	479 (0.97%)	*P* = 0.096	0.78 (0.58,1.05)
HELLP syndrome	1 (0.02%)	6 (0.01%)	*P* = 0.851	1.27 (0.15,10.57)
PPROM	2018 (31.30%)	16,808 (34.16%)	*P* < 0.001	0.88 (0.83,0.93)
Postpartum hemorrhage	178 (2.76%)	1151 (2.34%)	*P* = 0.037	1.19 (1.01,1.39)
GDM	1079 (16.74%)	7811 (15.87%)	*P* = 0.076	1.07 (0.99,1.14)
ICP	78 (1.21%)	161 (0.33%)	*P* < 0.001	3.73 (2.84,4.90)
Placental abruption	101 (1.57%)	537 (1.09%)	*P* < 0.001	1.44 (1.16,1.79)
Premature birth	669 (10.38%)	4546 (9.24%)	*P* = 0.003	1.14 (1.04,1.24)
Small for gestational age	265 (4.11%)	1817 (3.69%)	*P* = 0.096	1.12 (0.98,1.28)

Abbreviations: *GDM* Gestational diabetes, *ICP* Intrahepatic cholestasis of pregnancy, *PPROM* Preterm premature rupture of the membrane

### The association between HBV positive pregnancies and pregnancy outcomes by cesarean section

The results pertaining to the group of women who underwent cesarean section are listed in Table 4. Similar to that observed for the vaginal delivery group, the incidence of ICP in the HBsAg-positive group was higher than that in the HBsAg-negative group (OR, 3.06; 95% CI, 2.31–4.04). In contrast, the risk of the small-for-gestational-age outcome was significantly different, and the incidence of cesarean section was higher in the HBsAg-positive group compared with the HBsAg-negative group (OR, 0.68; 95% CI, 0.56–0.82). Moreover, there were no significant differences in premature rupture of membranes (OR, 0.91; 95% CI, 0.82–1.01), gestational hypertension (OR, 0.82; 95% CI, 0.64–1.05), HELLP syndrome (OR, 0.70; 95% CI, 0.25–1.95), GDM (OR, 1.03; 95% CI, 0.94–1.14), placental abruption (OR, 0.78; 95% CI, 0.56–1.08), preterm labor (OR, 0.91; 95% CI, 0.82–1.01), and postpartum hemorrhage (OR, 1.08; 95% CI, 0.77–1.50) between the groups.

**Table 4:** Gestational complications in Cesarean section

	HBV-positive (*n* = 3252)	HBV-negative (*n* = 23,870)	*P*	OR
Gestational hypertension	69 (1.48%)	617 (1.93%)	*P* = 0.115	0.82 (0.64, 1.05)
Preeclampsia	90 (2.77%)	823 (3.45%)	*P* = 0.044	0.80 (0.64, 0.99)
HELLP syndrome	4 (0.12%)	42 (0.18%)	*P* = 0.491	0.70 (0.25,1.95)
PPROM	559 (17.19%)	3968 (16.62%)	*P* = 0.416	1.04 (0.94,1.15)
Postpartum hemorrhage	40 (1.23%)	273 (16.62%)	*P* = 0.665	1.08 (0.77,1.50)
GDM	584 (17.95%)	4171 (17.47%)	*P* = 0.495	1.03 (0.94,1.14)
ICP	71 (2.18%)	173 (0.72%)	*P* < 0.001	3.06 (2.31,4.04)
Placental abruption	39 (1.20%)	368 (1.54%)	*P* = 0.132	0.78 (0.56,1.08)
preterm labor	489 (15.03%)	3878 (16.25%)	*P* = 0.078	0.91 (0.82,1.01)
Small for gestational age	127 (3.90%)	1348 (5.65%)	*P* < 0.001	0.68 (0.56,0.82)

Abbreviations: *GDM* Gestational diabetes, *ICP* Intrahepatic cholestasis of pregnancy, *PPROM* Preterm premature rupture of the membrane

## Discussion

Our study revealed an association between chronic HBV infection and an increased risk of placental abruption, preterm labor, ICP, GDM, SGA and postpartum hemorrhage. Moreover, we detected a decreased risk of premature rupture of membranes in women who underwent vaginal delivery, and of PIH regardless of delivery mode. However, no significant differences were observed between the group with chronic HBV infection and the control group regarding the prevalence of GDM, HELLP, and small-for-gestational-age status*.*

A higher prevalence of preterm delivery in women with chronic HBV infection was reported in several epidemiological studies [6, 7], which is consistent with our results. Inflammation is one of the most common risk factors for preterm labor [17, 18]. Therefore, it is plausible to hypothesize that low-grade inflammation induced by chronic HBV infection is partially responsible for the increased risk of preterm labor observed in these studies. Local and systemic inflammation caused by HBV infection may play an independent etiological role in preterm birth. According to previous studies, a large proportion of preterm births are caused by overproduction of proinflammatory cytokines [19]. The liver is a critical organ for human health, especially because it regulates inflammation by controlling local and systemic inflammatory responses via different molecular mechanisms [20]. However, chronic HBV infection can cause liver dysfunction, thus affecting inflammatory regulation. Another explanation for the association between maternal HBV infection and preterm birth is the accumulation of viral DNA in the placenta and in the trophoblast cells that initiate the placental inflammatory response, which is a known contributor to preterm birth [21]. Therefore, further research is needed to elucidate the mechanism underlying the association between preterm birth and HBV infection.

ICP is a pregnancy-specific liver disorder that typically commences in the late-second or third trimester and resolves within 48 h after delivery. We found that pregnant women with HBsAg positivity exhibited an increased risk of ICP compared with pregnant women who were HBsAg negative (0.46% versus 1.54%, *P* < 0.05). Therefore, HBV infection may also be related to an increased incidence of ICP, but further research of the underlying mechanism is warranted. Elevated estrogen levels are a possible cause of ICP. Because the liver is the main organ for estrogen metabolism, the dysregulation of liver function due to HBV infection may lead to metabolic disorders of estrogen. This is a possible cause of HBV-infection-induced ICP. Moreover, the risk of postpartum hemorrhage was increased in the HBV-infected group compared with the control group, which is a plausible outcome if liver function is affected.

We noticed that chronic HBV infection was associated with an increased risk of placental abruption. HBV infection may affect trophoblast cells, which play an important role in placental development [10, 11]. The HBV X protein may contribute to the migration and invasion of some types of cancer cells [22, 23]. Thus, it is plausible to hypothesize that the trophoblast migration and invasive capacity is also increased in women with chronic HBV infection. Moreover, subclinical inflammation caused by chronic HBV infection may induce trophoblast dysfunction. A recent publication demonstrated that inflammation decreases extravillontation and the associated placental trophoblast invasion [24], thus contributing to placental complications. This process causes abnormal implantation and impaired spiral artery remodeling, which are involved in the pathogenesis of placenta previa and placental abruption [11, 25]. Although Huang et al. reported no significant association between chronic HBV infection and an increased risk of placental abruption [26], our findings imply that chronic HBV infection increases the risk of this complication.

Two recent studies reported an independent association between chronic HBV infection and GDM [12, 13], which is in disagreement with our results. Lao et al. reported that the incidence of gestational diabetes was significantly increased in the HBsAg-positive group. Multiple logistic regression analysis revealed an independent association between HBsAg carrier status and GDM (relative risk, 3.51; 95% CI, 1.83–6.73) [27]. Liver plays a key role in regulating glucose homeostasis. Liver damage from the hepatitis B virus might cause a glycometabolic disorder [28], Therefore, additional studies are required to elucidate this issue.

Although the mechanism underlying the association between maternal HBV infection and the incidence of PIH is unclear, the latter is most likely related to an altered maternal immune response to the fetal allograft, toward increased immunotolerance, leading to a reduced incidence of PIH and PE. A multicenter study performed by Reddick et al. showed that maternal HBV infection was not associated with PE [6]. Another multicenter study carried out in Florida and a cohort study performed in Liuyang in rural China also reported an absence of significant associations between HBV infection and PIH [16, 29]. However, the authors of a case–control study that was carried out in Iran and included 450 HBV carriers and 450 controls reported an increased risk of PIH in women with HBV infection (OR, 54.2; 95% CI, 2.2–8.1) [30]. In contrast, Lao et al. found that maternal HBV infection reduced the risk of PIH (OR, 50.79; 95% CI, 0.66–0.95) and PE (OR, 50.71; 95% CI, 0.56–0.91) [4]. Here, maternal HBV infection reduced the risk of PIH (HbsAg-positive group: 3.05%; HBsAg-negative group: 3.73%). The inconsistency of these results may be explained by a selection bias caused by the research methods and the size of the cohorts used in each study.

Interestingly, there was a significant difference between the HBV-infected and the normal groups regarding mode of delivery. Here, a decreased risk of premature rupture of fetal membranes was observed in HBV-infected women compared with the normal group, whereas a previous report describes opposite findings [31]. These results suggest that HBV-infected pregnant women may maintain a good self-management level and obtain additional help from their physicians and families.

Preeclampsia (which is characterized by multilevel maternal endothelial dysfunction) may be triggered by or result from an imbalance between angiogenic, antiangiogenic, and proangiogenic factors, e.g., vascular endothelial growth factor [8, 14]. Moreover, significant associations/correlations between HBV, insulin resistance, obesity, and renal injury/proteinuria have been reported [32–34]. Lao et al. reported that the incidence of gestational diabetes was significantly increased in the HBsAg-positive group. Multiple logistic regression analysis revealed an independent association between HBsAg carrier status and GDM (relative risk, 3.51; 95% CI, 1.83–6.73) [27]. Cui et al. showed that the proportion of miscarriages was significantly higher among HBV carriers compared with controls (9.36% versus 5.70%; *P* < 0.001), which suggests that maternal HBV carrier status is an independent risk factor for miscarriage and that careful surveillance of these women is necessary [35]. Chronic HBV infection is a major cause of liver dysfunction (e.g., it increases serum aminotransferase and bilirubin levels). However, preterm birth was more common in women with abnormal alanine aminotransferase (ALT), aspartate aminotransferase (AST), glutamyl transpeptidase (GGT), or total bilirubin. Xun Zhuang et al. showed that the incidences of ICP, preeclampsia/eclampsia, and HELLP syndrome were 1.37, 3.14, and 0.02%, respectively [36].

A large-scale population-based cohort study showed that pre-pregnancy HBV infection may be associated with an increased risk of preterm delivery. Here, women who were HBsAg positive and HBeAg negative had a 26% higher risk of preterm birth, and women who were both HBsAg and HBeAg positive had a 20% higher risk of preterm birth than did women who were not infected with HBV. In addition, women who were HBsAg positive and HBeAg negative exhibited an 18% higher risk of early preterm birth, and women who were both HBsAg and HBeAg positive had a 34% higher risk of early preterm birth compared with women without HBV infection. Maternal pre-pregnancy HBV infection is independently associated with a higher risk of preterm birth and early preterm birth [37]. Moreover, three review articles reported a significantly increased risk of preterm birth among HBV carriers [35, 38, 39]. Safir et al. found that the presence of the HBsAg in pregnant women poses an additional risk for the pregnancy [6, 12, 21, 40, 41]; in contrast, Pastorek et al. stated that there is no significant difference between HBV-infected and non-infected pregnant women [5, 42].

It’s found that HBV-positive pregnant women underwent vaginal delivery were more likely to have placental abruption and premature birth compared with HBV-negative women in our study. Placental abruption, classically defined as a premature separation of the placenta before delivery, is one of the leading causes of vaginal bleeding in the second half of pregnancy. It’s proposed that changes in the population and function of immunocytes at the maternal-fetal interface can be part of the mechanism of disease of obstetrical disorders, such as placental abruption [43], and whether HBV would affect the population and function of immunocytes at the maternal-fetal interface was unclear, perhaps this was the reason why HBV-positive pregnant women underwent vaginal delivery were more likely to have placental abruption.

This study had several limitations. First, we collected very few data regarding viral load in the HBV carriers included in the analyses. Second, we did not examine HBV activity (e.g., `16yt55g4erwq32156rt2HBV DNA), which might affect pregnancy outcomes. Moreover, the presence of HBeAg in serum indicates active viral replication in hepatocytes; thus, it is a surrogate marker of HBV DNA [44]. Therefore, we were unable to conclude on the presence/absence of significant correlations between higher viral loads at the time of pregnancy and adverse outcomes. Third, potential confounders that might affect pregnancy outcomes were not included in this study. For example, cigarette smoking, and alcohol and caffeine consumption may be risk factors for miscarriage. In fact, in Chinese cultural tradition, women are discouraged from drinking alcohol and smoking cigarettes, and most people drink tea rather than coffee. Another limitation of this study was its single-center design: the study was carried out in a tertiary maternal and child health hospital; however, this hospital serves nearly half of the city’s population and guarantees good patient representativeness. Finally, this study lacked information regarding hepatitis B e-antigen status and liver function, further research should focus on the effect of hepatitis B e-antigen status on pregnancy outcomes.

## Conclusion

The present results suggest that compared with HBV positive pregnancies were more likely to be ICP and postpartum hemorrhage. HBV-positive pregnant women underwent vaginal delivery were more likely to have placental abruption abruption and premature birth compared with HBV-negative women. Obstetricians should be aware of ICP, postpartum hemorrhage, placental abruption and premature birth in HBV-positive pregnant women..

## Data Availability

The datasets used and analysed during the current study are available from the corresponding author on reasonable request.

## Abbreviations

HBV: Chronic hepatitis B viral
CHB: Chronic hepatitis B.
HBsAg: 1. hepatitis B surface antigen
MTCT: Mother-to-child transmission
PIH: Pregnancy-induced hypertension syndrome
GDM: Gestational diabetes
ICP: Intrahepatic cholestasis of pregnancy
SD: Standard deviation
