# Correction to: Maternal HBsAg carriers and pregnancy outcomes: a retrospective cohort analysis of 85,190 pregnancies

**DOI:** 10.1186/s12884-021-03630-x

**Published:** 2021-02-12

**Authors:** Yulong Zhang, Jiacheng Chen, Tingting Liao, Siwen Chen, Jianying Yan, Xiaoqian Lin

**Affiliations:** Department of Obstetrics and Gynecology, Fujian Provincial Maternity and Children’s Hospital, Affiliated Hospital of Fujian Medical University, Fuzhou, 350001 Fujian China

**Correction to: BMC Pregnancy Childbirth 20, 724 (2020)**

**https://doi.org/10.1186/s12884-020-03257-4**

Following publication of the original article [1], the authors reported an error in Tables 1, 2 and 3 headers. The correct tables are given below.

**Table 1:** Baseline characteristics of the included patients^a^

	HBV-positive (*n* = 9699)	HBV-negative (*n* = 73,076)	*P* value	OR
Maternal age (yrs, mean ± SD)	30.33 ± 4.50	30.28 ± 4.45	*P* = 0.30	
Gestational age (weeks, mean ± SD)	38.18 ± 2.96	38.17 ± 3.51	*P* = 0.76	
Gravidity				
1	3323 (34.26%)	28,076 (38.42%)	*P* < 0.001	0.84 (0.80,0.87)
> 1	6376 (65.74%)	45,000 (61.58%)	*P* < 0.001	1.20 (1.15,1.25)
Parity				
1	82 (0.85%)	760 (1.04%)	*P* = 0.07	0.81 (0.65,1.02)
> 1	9617 (99.15%)	72,316 (98.96%)	*P* = 0.07	1.23 (0.98,1.55)
Mode of delivery				
Instrumental vaginal	1903 (19.62%)	14,009 (19.17%)	*P* = 0.29	1.03 (0.98,1.09)
Cesarean section	3380 (34.85%)	24,729 (33.84%)	*P* = 0.05	1.05 (1.00,1.09)
Vaginal delivery	4373 (45.09%)	33,907 (46.40%)	*P* = 0.01	0.95 (0.91,0.99)
Regional analgesia	43 (0.44%)	431 (0.59%)	*P* = 0.07	0.75 (0.55,1.03)
Birth weight(g)				
< 3000	2855 (29.44%)	20,571 (28.15%)	*P* = 0.008	1.06 (1.02,1.122)
3000–3499	4026 (41.51%)	30,721 (42.04%)	*P* = 0.32	0.98 (0.94,1.02)
3500–3999	2231 (23.00%)	17,085 (23.38%)	*P* = 0.41	0.98 (0.93,1.03)
≥ 4000	388 (6.05%)	2718 (6.43%)	*P* = 0.17	1.08 (0.97,1.20)

**Table 2:** The association between HBV positive pregnancies and outcomes

	HBV-positive (*n* = 9699)	HBV-negative (*n* = 73,076)	*P* value	OR
Gestational hypertension	157 (1.62%)	1421 (1.94%)	*P* = 0.028	0.83 (0.70,0.98)
Preeclampsia	139 (1.43%)	1302 (1.78%)	*P* = 0.014	0.80 (0.67,0.96)
HELLP syndrome	5 (0.05%)	48 (0.07%)	*P* = 0.605	0.78 (0.31,1.97)
P*PROM*	2577 (26.57%)	20,776 (28.43%)	*P* < 0.001	0.91 (0.87,0.96)
Postpartum hemorrhage	218 (2.25%)	1424 (1.95%)	*P* = 0.047	1.16 (1.00,1.34)
GDM	1663 (17.15%)	11,982 (16.40%)	*P* = 0.062	1.06 (1.00,1.12)
ICP	149 (1.54%)	334 (0.46%)	*P* = 0.001	3.40 (2.80,4.13)
placental abruption	140 (1.44%)	905 (1.24%)	*P* = 0.089	1.17 (0.98,1.40)
Premature birth	1158 (11.94%)	8424 (11.53%)	*P* = 0.234	1.04 (0.97,1.11)
Small for gestational age	392 (0.22%)	3165 (0.19%)	*P* = 0.192	0.93 (0.84,1.04)

**Table 3:** Gestational complications in Vaginal delivery

	HBV-positive (*n* = 6447)	HBV-negative (*n* = 49,206)	*P*	OR
Gestational hypertension	88 (1.30%)	804 (1.41%)	*P* = 0.106	0.83 (0.67, 1.04)
Preeclampsia	49 (0.76%)	479 (0.97%)	*P* = 0.096	0.78 (0.58,1.05)
HELLP syndrome	1 (0.02%)	6 (0.01%)	*P* = 0.851	1.27 (0.15,10.57)
PPROM	2018 (31.30%)	16,808 (34.16%)	P < 0.001	0.88 (0.83,0.93)
Postpartum hemorrhage	178 (2.76%)	1151 (2.34%)	*P* = 0.037	1.19 (1.01,1.39)
GDM	1079 (16.74%)	7811 (15.87%)	*P* = 0.076	1.07 (0.99,1.14)
ICP	78 (1.21%)	161 (0.33%)	P < 0.001	3.73 (2.84,4.90)
Placental abruption	101 (1.57%)	537 (1.09%)	P < 0.001	1.44 (1.16,1.79)
Premature birth	669 (10.38%)	4546 (9.24%)	*P* = 0.003	1.14 (1.04,1.24)
Small for gestational age	265 (4.11%)	1817 (3.69%)	P = 0.096	1.12 (0.98,1.28)

Statement “YLZ and JCC contributed equally to this work.” in the Authors’ contributions section deleted. “Yulong Zhang and Jiacheng Chen contributed equally to this work.” was inserted as article note.

The original article [1] has been updated.
